# Supplementary figures and images for: Mucus Trail Proteins May Infer Reproductive Readiness for Land Snails
Source: Biology (Basel). 2025 Mar 14;14(3):294. doi: 10.3390/biology14030294 (PMC11940790; doi:10.3390/biology14030294)

MWM

Supernatant

Pellet

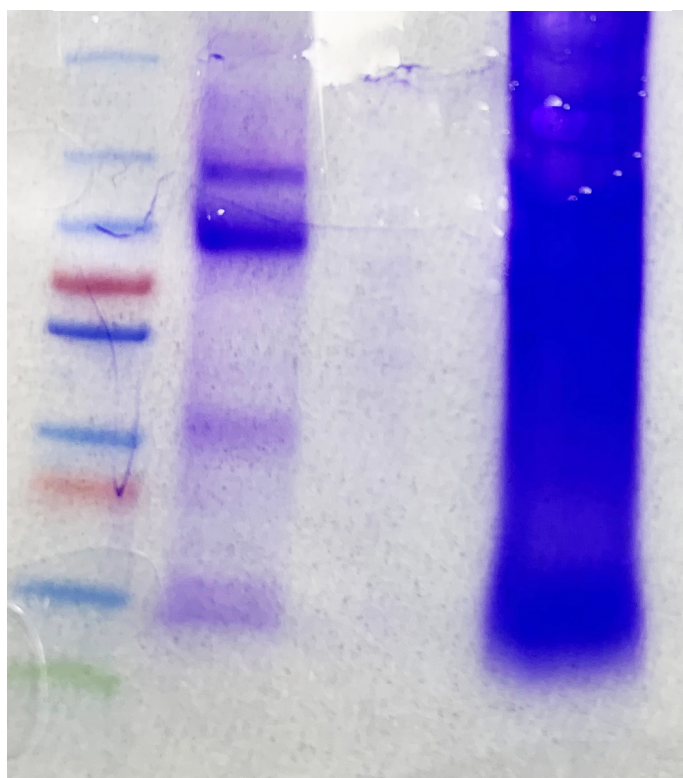

Supplement: Supplementary file 1 [file biology-14-00294-s001.zip › biology-3409329-supplementary Update/File S4-SDS-PAGE original Image.pdf]
